# Pathogenesis to Treatment: Preventing Preterm Birth Mediated by Infection

**DOI:** 10.1155/S1064744997000173

**Published:** 1997

**Authors:** James A. McGregor, Janice I. French

**Affiliations:** Department of Obstetrics and Gynecology University of Colorado Health Sciences Center 4200 East Ninth Avenue Campus Box B198 Denver CO 80262 USA

## Abstract

Prevention of preterm birth and subsequent newborn immaturity is a primary goal of obstetrical care worldwide. Accumulated evidence shows that 1) as many as 25–50% of preterm births are caused by common genital tract infections and subsequent maternal/fetal inflammatory responses; 2) microbial and maternal host factors (phospholipases, proteases, etc.) play roles in preterm labor and preterm premature rupture of membranes (pPROM); 3) integrated aspects of maternal and fetal host responses (inflammation, altered immune adaptations, endocrine and paracrine mechanisms) play increasingly understood roles in premature activation of parturition; and 4) identification and systemic treatment of common genitourinary infections, most importantly bacterial vaginosis (BV), reduce the risks of preterm delivery and PROM.


Infectious Diseases in Obstetrics and Gynecology 5:106–114 (1997)
© 1997 Wiley-Liss, Inc.

### Pathogenesis to Treatment: Preventing Preterm Birth Mediated by Infection

**James A. McGregor* and Janice I. French**
*Department of Obstetrics and Gynecology, University of Colorado Health Sciences Center, Denver, CO*

Abstract

**Prevention of preterm birth and subsequent newborn immaturity is a primary goal of obstetrical care worldwide. Accumulated evidence shows that 1) as many as 25–50% of preterm births are caused by common genital tract infections and subsequent maternal/fetal inflammatory responses; 2) microbial and maternal host factors (phospholipases, proteases, etc.) play roles in preterm labor and preterm premature rupture of membranes (pPROM); 3) integrated aspects of maternal and fetal host responses (inflammation, altered immune adaptations, endocrine and paracrine mechanisms) play increasingly understood roles in premature activation of parturition; and 4) identification and systemic treatment of common genitourinary infections, most importantly bacterial vaginosis (BV), reduce the risks of preterm delivery and PROM. Infect. Dis. Obstet. Gynecol. 5:106–114, 1997.** © 1997 Wiley-Liss, Inc.

Key words
prematurity; preterm birth; premature rupture of membranes; infection; inflammation; prevention; therapy; antibiotic; chorioamnionitis

"*Infection in the female reproductive tract (especially in the cervix) can cause premature rupture of membranes and induce premature labor. . . . This process is responsible for many preventable infant deaths.*[1]"

Gestational age at birth and birth weight are the most important biologic determinants worldwide of an individual child's chances of survival and healthy growth.[2] Immediate sequelae of biologic immaturity at birth include respiratory distress, intraventricular hemorrhage, leukomalacia, necrotizing enterocolitis, prolonged hospitalization, and death.[3,4] Among survivors, life-long complications can include cerebral palsy, cognitive impairment, blindness, and deafness.[3,4] The direct and indirect costs of biologic immaturity at birth can be immense. Estimates of the excess direct medical costs attributable to preterm infants totaled $6 billion U.S. in 1988.[5] The total individual, family, and societal burdens imposed by biologic immaturity at birth are beyond measure.

### BACTERIAL VAGINOSIS (BV) AND PRETERM BIRTH

The evidence for microbial causes of preterm birth is most extensive and convincing for BV. Nevertheless, there is continuing misapprehension that lower reproductive tract infections and BV are "mere markers" of upper tract intrauterine infection.[6] BV is not truly an infection, but rather a microecological condition in which there are dramatic alterations in the endogenous vaginal microflora. Hydrogen peroxide-producing *Lactobacillus* strains (including *L. jensenii* and *L. crispatus*) are reduced in number.[7,8] BV features multilog population increases in a characteristic set of microflora which includes *Gardnerella vaginalis,* genital anaerobes, and genital mycoplasmas.[7] These microbes, along

*Correspondence to: Dr. James A. McGregor, Department of Obstetrics and Gynecology, University of Colorado Health Sciences Center, 4200 East Ninth Avenue, Campus Box B198, Denver, CO 80262, E-mail: McGregoJ@Jove.UCHSC.edu

Received 1 October 1997

Accepted 21 October 1997

with coliforms and streptococci, are the same as found in most cases of chorioamnionitis.[9,10] BV is associated with increased vaginal and cervical fluid concentrations of endotoxin, proteases, mucinases, sialidases, IgA proteases, and phospholipases $A_2$ and C.[11–16] Observational studies show that the presence of BV early in pregnancy is associated with second trimester labor and perinatal loss or so-called late miscarriage.[6,17]

Multiple studies have been completed worldwide which demonstrate consistent associations of preterm birth with BV as well as our ability to reduce the risks with systemic (oral) treatment.[6,10,11,16–19] Table 1 displays findings of studies linking BV and preterm birth and/or premature rupture of membranes (PROM). Table 2 shows the results of intervention studies targeted at BV to reduce risks of prematurity as well as neonatal and maternal sequelae.

This and other information indicates that 1) BV is a direct cause of adverse pregnancy outcomes rather than a surrogate marker and 2) ascending infection or abnormal lower reproductive tract microflora mediate adverse pregnancy outcomes. Similar microbe-host interactions occur in periodontal disease and peptic ulcer disease, which are also effectively treated with antimicrobial agents, including metronidazole and clindamycin. How demographic, behavioral, obstetric, and infection factors interact with BV or other microbe-associated conditions is under continuing investigation. Figure 1 describes an "illness script" which depicts how some of these interactions may occur.

### MICROBIAL PATHOGENESIS

Microorganisms can act directly on reproductive tract tissues to initiate preterm labor and PROM or perpetuate these processes possibly begun by other factors. Microorganisms produce a variety of proteolytic enzymes including matrix metalloproteases, i.e., collagenases, elastases, IgA proteases, mucinases, and sialidases.[13–16,20–23] These pluripotential enzymes are involved in aspects of microbe pathogenesis including attachment, overcoming maternal host defenses, as well as directly impairing fetal membrane strength and elasticity.[11–16,20–23] Presence of enzymes such as sialidases, which facilitate bacterial attachment and break down mucin, may be required to facilitate attachment while mucinases may assist microbe ascent into uterine tissues.[16] Proteolytic enzymes may act directly on cervical collagen and amnion-chorion leading to premature cervical ripening and weakening of the fetal membranes with subsequent preterm PROM (pPROM).[20–22] Collagen biosynthesis may be disrupted by phospholipase $A_2$- or C-mediated prostaglandin release as well as specific microbial or host proteolytic enzymes.[13] Similarly, proteases may act as immunogenic agents and activate or amplify host inflammatory responses.[23]

Vaginal fluid levels of endotoxin, sialidases, phospholipase $A_2$ ($PLA_2$), prostaglandin $E_2$ ($PGE_2$), and interleukin-1 are greatly increased among women with BV.[11–16] Increased levels of vaginal $PLA_2$ are directly associated with increased risks of preterm labor and birth.[13] *Trichomonas vaginalis* also directly produces $PLA_2$, as well as a number of proteolytic enzymes in vitro.[23,24]

### HOST INFLAMMATORY PROCESSES

Dynamic maternal and fetal host inflammatory processes are of paramount importance in the pathogenesis of infection-mediated preterm birth and PROM. Multiple lines of cellular, animal model, and clinical investigation confirm evidence of inflammation in both maternal and fetal tissues in the pathogenesis of some but not all instances of prematurity. These findings include the presence of inflammatory mediators such as interleukin-1-β, interleukin-6, interleukin-8, and tumor necrosis factor-α (TNF-α) more often within the amniotic fluid of women with 1) amniotic fluid infection, 2) PROM, and 3) among women who continue preterm labor to delivery compared with women in whom preterm labor is successfully interrupted.[9,25–30] Recent investigations show that increased levels of interleukin-6 are detectable within cervical fluid of women with idiopathic preterm labor who have intraamniotic fluid infection and among women during antenatal care who subsequently deliver prior to 37 weeks gestation.[31,32]

In vitro and animal model studies confirm and inform clinical studies showing causal relationship between infection and preterm birth. Interleukin-1, interleukin-6, and TNF-α production have been demonstrated by cultured human decidual cells stimulated by bacterial products.[3,25,33] Similarly, amnion or decidual explants have been shown to produce prostaglandins in response to these cyto-

TABLE 1. Review of the literature evaluating specific microorganisms and PTB, LBW, or pPROM[a]

| Microorganism | Study design | Gestational age tested (weeks) | Positive | Negative | Outcome | Risk ratio (95% CI) |
|---|---|---|---|---|---|---|
| BV | | | | | | |
| Minkoff, 1984 | Prospective cohort | 13 | ND | ND | PTB | 2.3 (0.96–5.5)[b] |
| Gravett, 1986 | Prospective cohort | 32 | 24/102 (24.0%) | 65/432 (15.0%) | LBW | 1.7 (1.0–2.9) |
| | | | 22/102 (21.6%) | 44/432 (10.2%) | pPROM | 2.0 (1.1–3.7) |
| McDonald et al., 1991[10] | Prospective cohort | 22–28 | 31/135 (23%) | 97/651 (15%) | PTB | 1.8 (1.01–3.2) |
| | | | ND | ND | pPROM | 2.7 (1.1–6.5) |
| Kurki et al., 1992[18] | Prospective cohort | 8–17 | 11/162 (6.8%) | 6/571 (1.0%) | PTB | 6.9 (2.5–18.8) |
| | | | 6/162 (3.7%) | 3/571 (0.5%) | pPROM | 7.3 (1.8–29.4) |
| Joesoef, 1993 | Prospective cohort | 16–20 | 17/84 (20.2%) | 48/406 (11.8%) | PTB | 2.0 (1.0–3.9) |
| | | 28–32 | 11/67 (16.4%) | 50/395 (12.7%) | PTB | 1.5 (0.7–3.0) |
| McGregor et al., 1994[16] | Prospective cohort | 16–26 | 14/129 (10.9%) | 4/122 (3.3%) | PTB | 3.3 (1.2–9.1) |
| | | | 6/128 (4.7%) | 1/121 (0.1%) | pPROM | 5.7 (0.9–36.1) |
| Hay et al., 1994[6] | Prospective cohort | <16 | 7/57 (12.3%) | 9/384 (2.3%) | PTB | 5.2 (2.0–13.5) |
| | | <24 | 8/83 (9.6%) | 18/616 (2.9%) | PTB | 3.3 (1.5–7.4) |
| McGregor et al., 1995[17] | Prospective cohort | 18 | 31/165 (18.8%) | 37/380 (9.7%) | PTB | 1.9 (1.2–3.0) |
| | | | 10/144 (6.9%) | 7/350 (2.0%) | pPROM | 3.5 (1.4–8.9) |
| Meis et al., 1995[48] | Prospective cohort | 24 | ND | ND | PTB <35 weeks | 1.4 (0.9–2.0) |
| | | 28 | ND | ND | PTB <35 weeks | 1.8 (1.2–3.0) |
| Hillier et al., 1995[19] | Prospective cohort | 23–26 | 77/1,218 (6.3%) | 291/6,978 (4.2%) | PTB + LBW | 1.4 (1.1–1.8) |
| | | | 35/1,132 (3.1%) | 182/6,617 (2.8%) | pPROM | 1.1 (0.8–1.6) |
| *Trichomonas vaginalis* | | | | | | |
| Grice, 1974[45] | Retrospective chart review | ND | 17/748 (2.3%) | 76/7,482 (1.0%) | pPROM | 2.2 (1.3–3.7)[b] |
| Ross, 1983 | Prospective cohort | <34 | ND (12.0%) | ND (7.0%) | LBW | 1.7 (0.8–3.7)[b] |
| Hardy, 1984 | Prospective cohort | 13 | ND (18.0%) | ND (6.7%) | LBW | 2.6 (1.1–5.9)[b] |
| Joesoef, 1993 | Prospective cohort | 16–20 | ND | ND | PTB | 1.8 ND[b] |
| Read, 1993 | Prospective cohort | 23–26 | ND (15.4%) | ND (9.9%) | PTB | 1.4 (1.2–1.8)[c] |
| McGregor et al., 1995[16] | Prospective cohort | 18 | 8/50 (16.0%) | 56/460 (12.2%) | PTB | 1.6 (0.8–3.2) |
| | | | 3/45 (6.7%) | 14/418 (3.4%) | pPROM | 2.0 (0.6–6.7) |
| Cotch, 1990[44] | Prospective cohort | 23–26 | ND (14.8%) | ND (11.0%) | PTB | 1.3 (1.1–1.4) |
| | | | ND (4.0%) | ND (2.8%) | pPROM | 1.2 (0.9–1.5) |
| Meis et al., 1995[48] | Prospective cohort | 24 | ND | ND | PTB <35 weeks | 1.5 (0.1–1.8) |
| | | 28 | ND | ND | PTB <35 weeks | 0.9 (0.2–3.6) |
| *Chlamydia trachomatis* | | | | | | |
| Harrison, 1983 | Prospective cohort | <32 | 7/17 (41.2%) | 74/790 (9.4%) | pPROM | 4.4 (2.4–8.1)[b] |
| Gravett, 1986 | Prospective cohort | 13–42 | ND (32.0%) | ND (15.0%) | LBW | 2.7 (1.3–5.7) |
| | | | ND (23.0%) | ND (11.0%) | pPROM | 2.4 (1.1–5.4) |
| Sweet, 1987 | Prospective cohort | 1st visit | 22/304 (7.2%) | 433/6,242 (6.9%) | PTB | 1.05 (0.6–1.7)[b] |
| | | | 12/304 (3.9%) | 120/6,242 (1.9%) | pPROM | 2.0 (1.2–3.7)[b] |
| McGregor et al., 1990[38] | Prospective cohort | 22–29 | ND | ND | PROM | 2.5 (1.1–5.7) |
| Ryan et al., 1990[41] | Prospective cohort | 1st visit | 218/1,110 (19.6%) | 1,068/9,111 (11.7%) | LBW | 1.7 (1.5–1.9) |
| | | | 58/1,110 (5.2%) | 243/9,111 (2.7%) | pPROM | 2.1 (1.6–2.9) |
| Cohen et al., 1990[42] | Prospective cohort | 22–30 | 16/79 (20.2%) | 18/244 (7.3%) | PROM | 2.8 (1.5–5.1) |
| Martin et al., 1990[43] | Prospective cohort | 23–26 | 28/520 (5.4%) | 67/2,250 (3.0%) | pPROM | 1.8 (1.2–2.8) |
| Donder, 1993 | Prospective cohort | 1st visit | 6/22 (27%) | 23/145 (16%) | PTB | 2.0 (0.6–6.1) |
| *Neisseria gonorrhoeae* | | | | | | |
| Amtsey, 1976 | Retrospective chart review | ND | 56/198 (28.3%) | 557/4,246 (13.1%) | PTB | 2.6 (1.9–3.6)[b] |
| Edwards, 1978 | Prospective matched pair | ND | 8/19 (42.1%) | 5/41 (12.2%) | PTB | 5.2 (1.2–23.8) |
| Donders, 1993 | Prospective cohort | 1st visit | 5/9 (56.0%) | 24/158 (15.0%) | PTB | 6.0 (1.5–34.0) |
| Elliott, 1990 | Case control | At delivery | ND (11.2%) | ND (3.8%) | PTB | 5.3 (1.6–17.9) |

[a]PROM = preterm premature rupture of membranes; PTB = preterm birth <37 weeks gestation; LBW = low birth weight; ND = not described.
[b]Risk ratio and 95% confidence intervals calculated from data provided.
[c]Group with frequent intercourse and *T. vaginalis* vs. no *T. vaginalis*.

TABLE 2. Literature review which examined treatment for specific infections and PTB[a]

| Microorganism | Study design | Subjects | Outcome | Treated | Control | Risk ratio (95% CI) |
|---|---|---|---|---|---|---|
| *N. gonorrhoeae* | | | | Treated | Untreated | |
| Charles, 1970 | Open retrospective comparison | ND | PROM | 4/144 (2.8%) | 6/14 (43%) | 0.06 (0.02–0.2)[b] |
| *C. trachomatis* | | | | Erythromycin | Placebo | |
| Martin et al., 1990[43] | RCT, double blind placebo | All | LBW | 7/89 (7.9%) | 18/85 (21.2%) | 0.37 (0.16–0.84)[b] |
| | | | PTB | 9/89 (10.1%) | 17/85 (20%) | 0.51 (0.24–1.07)[b] |
| | | | pPROM | 1/87 (1.1%) | 6/81 (7.4%) | 0.16 (0.02–1.26)[b] |
| | | | | Treated | Untreated | |
| Ryan et al., 1990[41] | Clinical trial, control observational control | All | LBW | 145/1,323 (11.0%) | 218/1,110 (19.6%) | 0.56 (0.46–0.68)[b] |
| | | | pPROM | 39/1,323 (2.9%) | 58/1,110 (5.2%) | 0.56 (0.38–0.84)[b] |
| | | | | Erythromycin | Untreated | |
| Cohen et al., 1990[42] | Open retrospective comparison | All | PTB | 7/244 (2.9%) | 11/79 (13.9%) | 0.16 (0.06–0.47)[b] |
| BV | | | | Treated | Untreated | |
| Hillier et al., 1995[19] | Observation | All | PTB + LBW | 8/187 (4.3%) | 77/1,218 (6.3%) | 0.68 (0.33–1.38)[b] |
| | | | | Treated | Untreated | |
| Meis et al., 1995[48] | Observation | All | PTB <35 weeks | ND | ND | 0.44 (0.11–1.9) |
| | | | | Clindamycin | No treatment | |
| McGregor et al., 1995[17] | Clinical trial, observational control | All | PTB | 18/183 (9.8%) | 31/165 (18.8%) | 0.5 (0.3–0.9) |
| | | | pPROM | 6/171 (3.5%) | 10/144 (6.9%) | 0.5 (0.2–1.4) |
| | | | | Metronidazole | Placebo | |
| Morales et al., 1994[49] | RCT, double blind placebo | Prior PTB | PTB | 8/44 (18.0%) | 16/36 (39.0%) | 0.41 (0.2–0.8) |
| | | | pPROM | 2/44 (4.5%) | 12/36 (33.3%) | 0.14 (0.03–0.57) |
| | | | | Metronidazole | Placebo | |
| McDonald et al., 1996[50] | RCT, double blind placebo | All | PTB | ND (5.1%) | ND (6.4%) | 0.8 (0.3–1.8) |
| | | Prior PTB | PTB | 2/20 (10%) | 10/24 (42%) | 0.16 (0.02–0.9) |
| | | | | Metronidazole + erythromycin | Placebo | |
| Hauth et al., 1995[47] | RCT, double blind placebo | Prior PTB or <50 kg | PTB | 54/172 (31%) | 42/86 (49%) | 0.6 (0.5–0.9) |
| *T. vaginalis* | | | | Metronidazole | No treatment | |
| Morgan, 1978 | Observation | All | LBW | 10/597 (17.5%) | 53/283 (18.5%) | 0.95 (0.7–1.28)[b] |
| | | | PTB | 32/597 (5.3%) | 13/283 (4.5%) | 1.17 (0.6–2.19)[b] |
| | | | | Treated | No treatment | |
| Ross, 1983 | Observation | All | LBW | ND (12.0%) | ND (11.0%) | ND |
| | | | | Metronidazole | No treatment | |
| McGregor et al., 1995[17] | Clinical trial, observational control | All | PTB | 7/48 (14.6%) | 8/50 (16.0%) | 0.9 (0.34–2.3)[b] |
| | | | pPROM | 2/43 (4.6%) | 3/45 (6.7%) | 0.5 (0.2–1.4)[b] |

[a]See Table 1 for abbreviations. RCT =.
[b]Risk ratio and 95% confidence intervals calculated from data provided.

kines and endotoxin.[9,33] The work of Gravett et al.[34,35] with a monkey model of amniotic fluid infection demonstrates sequential increases in amniotic fluid concentrations of TNF-α, interleukin-6, interleukin-1-β, interleukin-1 receptor antagonists, $PGE_2$ and $F_{2\alpha}$ following intraamniotic inoculation of group B streptococcus. Furthermore, onset of uterine contractions ultimately leading to preterm labor and delivery occurred sequentially approximately 10 h following detection of rising amniotic fluid levels of interleukin-1 and parallel rises of prostaglandins $PGE_2$ and $F_{2\alpha}$.[34,35] Subsequent work with this model demonstrates TNF-α, $PGE_2$ and $F_{2\alpha}$ following infusion of interleukin-1-β in the absence of microorganisms.[36] Levels of TNF-α and interleukin-1-β were cleared within 48 h; however, prostaglandin levels remained elevated.[36] These and other cytokines directly contribute to increased levels of uterotonic prostaglandins.[36] Such cytokines may also contribute to the onset of

| | |
|---|---|
| • Enabling conditions | 1. Absence protective Lactobacilli<br>2. Sexual exposure to partner with "BV set" of microbes<br>3. Genetic predisposition |
| • Predisposing factors | 1. First or second trimester bleeding<br>2. Short or funneled cervix<br>3. Increased uterine activity<br>4. Increased vaginal fluid $PLA_2$, proteases<br>5. Concomitant reproductive tract infections |
| • Boundary conditions | 1. Black race, age |
| • Proximate cause/fault | 1. Invasion intrauterine tissues by microorganisms<br>2. Inflammatory response, maternal-fetal tissues |
| • Consequences | 1. Preterm labor<br>2. Preterm premature rupture of membranes |
| • Interventions | 1. Systemic antibiotic treatment: metronidazole, clindamycin, others<br>2. Reduce uterine contractions |
| • Results of interventions | 1. Reduced preterm birth, pPROM<br>2. Reduced direct, indirect costs<br>3. Reduced long-term morbidity |

Fig. 1. "Illness script" for BV-mediated preterm birth/PROM.

preterm birth by induction of matrix metalloproteases, which in turn enhance cervical ripening and weakening of the amnionchorion.[37]

Experiments performed with human fetal membranes show that substances produced by both microbes and host inflammatory cells have been shown to mediate prostaglandin release and membrane weakening in vitro.[21,22] Importantly, addition of appropriate antimicrobials including erythromycin and metronidazole during in vitro experiments of fetal membrane tensile strength prevents bacteria-induced fetal membrane weakening.[28,38]

Choice of antibiotics to treat infectious agents in both in vitro and in vivo and clinical investigations regarding preterm birth is crucial: agents such as clindamycin, erythromycin, aminoglycosides, and metronidazole tend to shut down bacterial virulence factor production. Conversely, beta lactam antibiotics (penicillins, cephalosporins) act primarily by impairing cell wall synthesis, allowing for increased local tissue release of bacterial cell constituents, including endotoxins [lipopolysaccharides (LPS)]. Use of bactericidal antibiotic agents may "throw gasoline" on the "fire of inflammation" and thus worsen outcomes. Further understanding of the nature and timing of microbe-maternal and fetal interactions and how best to interfere with these processes will lead to development of improved regimens for both prevention and treatment of preterm birth mediated by reproductive tract infection and inflammation.

### OBSTETRICAL STUDIES: CHLAMYDIA, GONORRHEA, AND TRICHOMONIASIS

Past observation and anecdotal studies (Table 1) show that treatment for *Neisseria gonorrhoeae* and *Chlamydia trachomatis* provides benefits in terms of reduced rates of preterm birth and PROM as well as prevention of ophthalmia neonatorum (Table 2).[39–42] Retrospective comparisons of women who received antenatal treatment for *N. gonorrhoeae* compared with untreated women demonstrated significant reductions of preterm birth and rupture of membranes.[39] Ryan and colleagues[41] reported significant reductions in the rate of low birth weight infants [odds ratio (OR) 0.56, 95% confidence interval (CI) 0.46–0.68] born to women who received antenatal treatment for *C. trachomatis* compared with retrospective, untreated control women. Similarly, Cohen and colleagues[42] described an 84% (OR 0.16, 95% CI 0.06–0.47) reduction in the rate of preterm birth among successfully treated, chlamydia-positive women compared with a retrospective control group of untreated chlamydia-positive women. Among results presented from the Vaginal Infections and Prematurity (VIP) study, Martin et al.[43] reported that among women enrolled at the New Orleans site, low birth weight (erythromycin treated 7.9% vs. placebo treated 21.2%, $P = 0.01$) and PROM (erythromycin treated 1.2% vs. placebo treated 7.4%, $P = 0.04$) were each significantly reduced among chlamydia-positive women who received erythromycin compared to women who received placebo. The VIP study also showed a 40% increased risk of low birth weight and prematurity if subjects suffered trichomoniasis.[44] Results of this large study confirm the role of *Trichomonas vaginalis* in preterm birth shown in prior smaller investigations.[45,46]

### INTERVENTION STUDIES VS. BV TO REDUCE RISKS OF PRETERM LABOR AND RUPTURE OF MEMBRANES

Recently published controlled trials demonstrate that important proportions of preterm births can be prevented in women considered to be at both

"high" or "normal" risk for preterm birth by screening for and treating asymptomatic BV in pregnancy (Table 2). Large studies by Hillier et al.[19] and Hauth et al.[47] add conclusive weight to well over a dozen prior studies linking BV and prematurity. Hillier et al.[19] reported the most recently analyzed portion of the large, multicenter VIP study of over 10,000 U.S. women. There was a 40% increase in low birth weight in women with asymptomatic, untreated BV.[19] Treatment with agents effective against BV (metronidazole) eliminated this excess risk.[19] Meis et al.[48] noted a similar finding in an observational study in North Carolina.

Hauth and colleagues[47] performed a placebo-controlled intervention trial using a 7 day course of oral metronidazole (500 mg twice daily) and enteric-coated erythromycin (300 mg twice daily) in women judged to be at increased risk of preterm birth owing to a prior history of short gestation or maternal low body mass (<50 kg). This combined regimen reduced risks of preterm birth in subjects with asymptomatic BV by approximately one-third in both risk groups.[47] In a subsequent analysis, treated women with no other identified causes of preterm birth had risks of PROM reduced by approximately 70% (Hauth, personal communication).

Such dramatic findings confirm those of a 1994 investigation by Morales et al.[49] in which women with both a history of prior preterm birth and findings of BV in the studied pregnancy were treated with either oral metronidazole or placebo. Morales et al.[49] similarly obtained approximately 70% reductions for prematurity, low birth weight, hospital admissions for preterm labor, and PROM.

Most recently, results from a randomized placebo-controlled treatment trial of two 1 g oral doses of metronidazole vs. placebo among Australian women with heavy growth (3–4+) of *Gardnerella vaginalis* isolated from the vagina (as a surrogate for BV) demonstrated a two-thirds reduction in the rate of preterm birth among women who had had a prior preterm birth and 35% reduction among women without this history.[50] In each of these cited studies, the beneficial effects were demonstrated in asymptomatic women; symptomatic subjects were treated outside of these protocols and eliminated from analysis.[47,49,50]

We conducted a large controlled, prospective evaluation of "screening and treating" prevalent infections in order to reduce preterm birth in Denver, CO.[17] We demonstrated that "routine" screening and systemic treatment of prevalent reproductive tract infections, including BV, in pregnancy reduces the occurrence of preterm birth and PROM by approximately 50% (1,260 patients, intent to treat analysis).[17] Treatment of BV with 300 mg oral clindamycin twice daily for 7 days reduced idiopathic preterm birth by 70%; there was a single instance of severe diarrhea.[17] Combination of BV and other prevalent infections such as trichomoniasis and/or common obstetric complications such as first trimester bleeding increased preterm birth to more than 30%; treatment with Centers for Disease Control (CDC)-recommended systemic (oral or intramuscular) treatments reduced risks of preterm birth by half in these multiply infected women (Table 3).

### COST BENEFIT/SAVINGS ANALYSIS

Recent economic models of preterm birth prevention focus on "added costs" of diagnosis and treatment of BV during pregnancy rather than economic savings obtained from preventing preterm birth.[51] Using conservative assumptions (585,000 cases of BV in U.S. pregnant women in 1993; costs of generic antibiotic treatment associated to be $29 U.S.), Oleen-Burkey and Hillier[51] estimated direct savings by preventing preterm birth caused by BV of $150 million annually. In this study, costs for diagnosis were assumed to be $20 U.S. for evaluating Amsel's clinical criteria or a gram stain of vaginal fluid.[51] Bloom and Lee[52] calculated a 25:1 direct cost savings for identifying and treating BV among "high risk" women. Neither of these models takes into account costs incurred after neonatal discharge or increased liability costs owing to avoidable adverse pregnancy outcomes.[5]

Other genitourinary tract infections such as chlamydia endocervicitis, trichomoniasis, and gonorrhea as well as asymptomatic bacteriuria are already sought out and treated as "standard of care" in most U.S. centers. Partners of pregnant women with sexually transmitted diseases should be treated and tests of cure should be performed as appropriate. Tests of cure for infected pregnant women and their partners with sexually transmitted disease are more urgent during pregnancy so as to 1) maximally reduce risks of preterm birth and 2) eliminate risks of neonatal ophthalmia and pneumonitis caused by "family pathogens." These costs are already provided for in contemporary obstetric care.

### CONCLUSIONS

Preterm birth continues as an urgent international health priority as well as a widely accepted measure of effective health delivery. Children born with the biologic disadvantages of prematurity necessitate intensive care and expenditures of immense human and economic resources. Surviving children frequently lead lives of diminished personal and economic potential. Clearly, the morbidity and excess costs caused by preterm birth are better prevented or mitigated prior to birth than dealt with in intensive care settings, specialized schools, and sustaining social programs.

On the basis of epidemiologic, clinical, microbiologic, and biochemical evidence, there is now adequate evidence that 1) reproductive tract infection and subsequent inflammation cause significant numbers of women to suffer preterm labor, pPROM, and preterm birth; 2) these adverse effects are caused by infection and inflammation of upper reproductive tract organs and tissues (i.e., placenta, decidua, amnionchorion); 3) the majority of these infections arise from lower reproductive tract sources including BV, cervicitis, or abnormal colonization (enteropharyngeal pathogens, including coliforms); and 4) many of these preterm births are preventable with prompt diagnosis and systemic (oral) antibiotic treatment during pregnancy. Future studies will focus on more accurately defining women and babies at risk, elucidating pathogenic interactions between microbes and both maternal and fetal hosts, and refining diagnostic and treatment strategies so as to provide maximum benefits while incurring minimal adverse effects and costs.

Medical care providers now have new opportunities and obligations to prevent as many infection-mediated births as possible by identifying and treating prevalent reproductive tract infections in their patients. We wager that optimal approaches will be shown to include identification and treatment of susceptible women prior to pregnancy, as part of preconceptual counseling and care.

### REFERENCES

1. Knox IC, Hoerner JK: The role of infection in premature rupture of the membranes. Am J Obstet Gynecol 59:190–194, 1950.
2. Berkowitz GS, Papiernik E: Epidemiology of preterm birth. Epidemiol Rev 15:414–443, 1993.
3. Saigal S, Feeny D, Furlong W, et al.: Comparison of the health-related quality of life of extremely low birth weight children and a reference group of children at age eight years. J Pediatr 125:418–425, 1994.
4. Hack M, Taylor HG, Klein N, et al.: School-age outcomes in children with birth weights under 750 g. N Engl J Med 331:753–759, 1994.
5. Lewit EM, Schuuman Baker L, Corman H, et al.: The direct cost of low birth weight. The future of children. Low Birth Weight 5:35–56, 1995.
6. Hay PE, Lamont RF, Taylor-Robinson D, et al.: Abnormal bacterial colonization of the genital tract and subsequent preterm delivery and late miscarriage. Br Med J 308:295–298, 1994.
7. Spiegel CA: Bacterial vaginosis. Clin Microbiol Rev 4: 485–502, 1991.
8. Giorgi A, Torriani S, Dellaglio F, et al.: Identification of vaginal lactobacilli from asymptomatic women. Microbiologica 10:377–384, 1987.
9. Hillier SL, Witkin SS, Krohn MA, et al.: The relationship of amniotic fluid cytokines and preterm delivery, amniotic fluid infection, histologic chorioamnionitis, and chorioamnion infection. Obstet Gynecol 81:941–948, 1993.
10. McDonald HM, O'Loughlin JA, Jolley P, et al.: Vaginal infection and preterm labour. Br J Obstet Gynaecol 98: 427–435, 1991.
11. Platz-Christensen JJ, Mattsby-Baltzer I, Thomsen P, et al.: Endotoxin and interleukin-1 in the cervical mucus and vaginal fluid of pregnant women with bacterial vaginosis. Am J Obstet Gynecol 169:1161–1166, 1993.
12. Platz-Christensen JJ, Brandberg A, Wiqvist N: Increased prostaglandin concentrations in the cervical mucus of pregnant women with bacterial vaginosis. Prostaglandins 43:133–141, 1992.
13. McGregor JA, French JI, Jones W, et al.: Association of cervico/vaginal infections with increased vaginal fluid phospholipase $A_2$ activity. Am J Obstet Gynecol 167: 1588–1594, 1992.
14. Glasson JH, Woods WH: Immunoglobulin proteases in bacteria associated with bacterial vaginosis. Aust J Med Lab Sci 9:63–65, 1988.
15. Kapatais-Zoumbos K, Chandler DKF, Barile MF: Survey of immunoglobulin A protease activity among selected species of *Ureaplasma* and *Mycoplasma:* Specificity for host immunoglobulin A. Infect Immun 47:704–709, 1985.
16. McGregor JA, French JI, Jones W, et al.: Bacterial vaginosis is associated with prematurity and vaginal fluid sialidase: Results of a controlled trial of topical clindamycin cream. Am J Obstet Gynecol 170:1048–1060, 1994.

17. McGregor JA, French JI, Parker R, et al.: Prevention of premature birth by screening and treatment for common genital tract infections: Results of a prospective controlled evaluation. Am J Obstet Gynecol 173:157–167, 1995.
18. Kurki T, Sivonen A, Renkonen O-V, et al.: Bacterial vaginosis in early pregnancy and pregnancy outcome. Obstet Gynecol 80:173–177, 1992.
19. Hillier SL, Nugent RP, Eschenbach DA, et al., for Vaginal Infections and Prematurity Study Group: Association between bacterial vaginosis and preterm delivery of a low-birth-weight infant. N Engl J Med 333:1737–1742, 1995.
20. McGregor JA, Lawellin D, Franco-Buff A, et al.: Protease production by microorganisms associated with reproductive tract infection. Am J Obstet Gynecol 154:109–114, 1986.
21. McGregor JA, French JI, Lawellin D, et al.: Bacterial protease-induced reduction of chorioamniotic membrane strength and elasticity. Obstet Gynecol 69:167–174, 1987.
22. Schoonmarker JN, Lawellin DW, Lunt B, et al.: Bacteria and inflammatory cells reduce chorioamniotic membrane integrity and tensile strength. Obstet Gynecol 74: 590–596, 1989.
23. Alderete JF, Newton E, Dennis C, et al.: Antibody in sera of patients infected with *Trichomonas vaginalis* is to trichomonad proteinases. Genitour Med 67:331–334, 1991.
24. Draper D, Jones W, Heine RP, et al.: *Trichomonas vaginalis* weakens human amniochorion in an in vitro model of premature membrane rupture. Infect Dis Obstet Gynecol 2:267–274, 1995.
25. Romero R, Mazor M, Brandt F, et al.: Interleukin-1-a and interleukin-1-b in preterm and term human parturition. Am J Reprod Immunol 27:117–123, 1992.
26. Romero R, Brody DT, Oyarzun E, et al.: Infection and labor. III. Interleukin-1: A signal for the onset of parturition. Am J Obstet Gynecol 160:1117–1123, 1989.
27. Romero R, Manogua KR, Mitchell MD, et al.: Infection and labor. IV. Cachectin-tumor necrosis factor in the amniotic fluid of women with intraamniotic infection and preterm labor. Am J Obstet Gynecol 161:336–341, 1989.
28. Romero R, Avila C, Santhanam U, et al.: Amniotic fluid interleukin-6 in preterm labor: Association with labor. J Clin Invest 85:1392–1400, 1991.
29. Allbert JR, Naaf RW III, Perry KG Jr, et al.: Amniotic fluid interleukin-6 and interleukin-8 levels predict the success of tocolysis in patients with preterm labor. J Soc Gynecol Invest 1:264–268, 1994.
30. Coultrip LL, Lien JM, Gomez R, et al.: The value of amniotic fluid interleukin-6 determination in patients with preterm labor and intact membranes in the detection of microbial invasion of the amniotic cavity. Am J Obstet Gynecol 171:901–911, 1994.
31. Lockwood CJ, Ghidini A, Wein R, et al.: Increased interleukin-6 concentrations in cervical secretions are associated with preterm delivery. Am J Obstet Gynecol 171:1097–1102, 1994.
32. Rizzo G, Capponi A, Riondaldo D, et al.: Interleukin-6 concentrations in cervical secretions identify microbial invasion of the amniotic cavity in patients with preterm labor and intact membranes. Am J Obstet Gynecol 175: 812–817, 1996.
33. Mitchell MD, Trautman MS, Dudley DJ: Cytokine networking in the placenta. Placenta 14:249–275, 1993.
34. Gravett MG, Witkin SS, Haluska GJ, et al.: An experimental model for intraamniotic infection and preterm labor in rhesus monkeys. Am J Obstet Gynecol 171: 1660–1667, 1994.
35. Gravett MG, Witkin SS, Novy MJ: A nonhuman primate model for chorioamnionitis and preterm labor. Semin Reprod Endocrinol 12:246–262, 1994.
36. Baggia S, Gravett MG, Witkin SS, et al.: Interleukin-1-β intra-amniotic infusion induces tumor necrosis factor-a, prostaglandin production, and preterm contractions in pregnant rhesus monkeys. J Soc Gynecol Invest 3:121–126, 1996.
37. So T, Ito A, Sato T, et al.: Tumor necrosis factor-α stimulates the biosynthesis of matrix metalloproteinases and plasminogen activator in cultured human chorionic cells. Biol Reprod 46:772–778, 1992.
38. McGregor JA, Schoonmaker JN, Lunt BD, et al.: Antibiotic inhibition of bacterially induced fetal membrane weakening. Obstet Gynecol 76:124–128, 1990.
39. Charles AG, Cohen S, Kass MB, et al.: Asymptomatic gonorrhea in prenatal patients. Am J Obstet Gynecol 57:479–482, 1981.
40. Temmerman M, Njagi E, Nagelkerke N, et al.: Mass antimicrobial treatment in pregnancy: A randomized, placebo-controlled trial in a population with high rates of sexually transmitted disease. J Reprod Med 210:176–180, 1995.
41. Ryan GM Jr, Abdella TN, McNeeley SG, et al.: *Chlamydia trachomatis* infection in pregnancy and effect of treatment on outcome. Am J Obstet Gynecol 162:34–39, 1990.
42. Cohen I, Veille J-C, Calkins BM: Improved pregnancy outcome following successful treatment of chlamydial infection. JAMA 263:3160–3163, 1990.
43. Martin DH, and VIP Study Group: Erythromycin treatment of *Chlamydia trachomatis* infection during pregnancy. Abstract 683. Presented at the 30th Annual Interscience Conference on Antimicrobial Agents and Chemotherapy, Atlanta, GA, October 21–24, 1990.
44. Cotch MF, for Vaginal Infections and Prematurity Study Group: Carriage of *Trichomonas vaginalis* is associated with adverse pregnancy outcome. Abstract 681. Presented at the 30th Annual Interscience Conference on Antimicrobial Agents and Chemotherapy, Atlanta, GA, October 21–24, 1990.
45. Grice AC: Vaginal infection causing spontaneous rupture of the membranes and premature delivery. Aust NZ J Obstet Gynecol 14:156–158, 1974.
46. Hardy PH, Hardy JB, Nell EE, Nell EE, Graham DA,

Spence MR, Rosenbaum RC: Prevalence of six sexually transmitted disease agents among pregnant inner-city adolescents and pregnancy outcome. Lancet 2:333–337, 1984.

47. Hauth JC, Goldenberg RL, Andrews WW, DuBard MB, Cooper RL: Reduced incidence of preterm delivery with metronidazole and erythromycin in women with bacterial vaginosis. N Engl J Med 333:1732–1736, 1995.
48. Meis PF, Goldenberg, RI, Mercer B, et al., and NICHD-MFMU Network: The preterm prediction study: Significance of vaginal infections. Am J Obstet Gynecol 173:1231–1235, 1995.
49. Morales WJ, Schorr S, Albritton J: Effects of metronidazole in patients with preterm birth in preceding pregnancy and bacterial vaginosis: A placebo-controlled, double-blind study. Am J Obstet Gynecol 171:345–349, 1994.
50. McDonald HM, O'Loughlin J, Vigneswaran R, et al.: Reduction in preterm birth by treatment of bacterial vaginosis. Presented at the 4th International Symposium on Bacterial Vaginosis, London, England, October 31–November 3, 1996.
51. Oleen-Burkey MA, Hillier SL: Pregnancy complications associated with bacterial vaginosis and their estimated costs. Infect Dis Obstet Gynecol 3:149–157, 1995.
52. Bloom BS, Lee DW: Costs of preventing preterm birth (letter). N Engl J Med 331:1338, 1996.
53. Schmidt HG, Norman GR, Boshulzen HPA: A cognitive perspective on medical expertise: Theory and implications. Acad Med 65:611–621, 1990.
